# A Practical Treatise on the Physical Exploration of the Chest, and the Diagnosis of Diseases Affecting the Respiratory Organs

**Published:** 1867-02

**Authors:** 


*A Practical Treatise on the Physical Exploration of the Chest, and the Diagnosis of Diseases affecting the Respiratory Organs.* By Austin Flint, M. D., Professor of the Principles and Practice of Medicine in the Bellevue Hospital Medical College, and in the Long Island Hospital: Fellow of the New York Academy of Medicine, etc. Second edition, revised. Philadelphia: Henry C. Lea, 1866.

Of all the Physicians in America, with whom we have any acquaintance, (and we only know him by his writings)

Dr. Flint is the man after our own heart, whom we would select as a model for the accomplished physician. Not that he is possessed of the brilliancy of genius that dazzels the world by the splendor of its eloquence; not that he rules the minds of his contemporaries like Rush, or Chapman, or Dewes; not that he is looked up to as the great intellectual luminary of his age, for the theories and opinions that shall rule the minds of medical men of his own time and for centuries to come. It is not for these nor for many other reasons we might mention that we admire Dr. Flint: the time for the worship of such attributes in the medical man has passed. The medical mind of the present day needs no master. The genius of medicine in our time has emancipated itself from the dictation of authority, and launches forth in the noon-day sun of scientific observation for its lessons of truth and wisdom.

Let us not be misunderstood. We have no desire to derogate from the God-given attributes of genius. The mastery of mind has been acknowled in all ages, and will continue to control the minds of men for all time to come. What we maintain is this: that it is not the transcendent intellect with its brilliant theories, its eloquent rhetoric, and specious argument, that leads in the science and art of medicine at the present day. Hippocrates and Galen, Sydenham and Cullen, Gregory and Rush, were good enough in their time—their age and generation deserve well of them—their towering minds illumined the darkness by which they were surrounded. Galen, alone, exercised undisputed authority in medicine for more than a thousand years. Our profession has entered upon a new epoch: it has cast off the swaddling clothes of subserviency to any one or dozen minds; and we conceive it impossible that any one mind, however much endowed, can exert any degree of supremacy in the science and art of medicine as now cultivated.

The laborious chemist in his laboratory; the patient observer at the microscope; the zealous cultivator of experimental physiology; the indefatigable pathologist in the

Dead House; the careful observer and recorder of facts at the bed-side: these are the authorities that now rule in medicine, and not the brilliant theorist nor bold speculator.

It is as the type of the true and progressive medical philosopher that we desire to present Professor Flint to our readers. In the invaluable work before us, we have a book of *facts* of nearly 600 pages, admirably arranged, clear, thorough, and lucid on all points, without prolixity; exhausting every point and topic touched; a monument of patient and long continued observation, which does credit to its author, and reflects honor on American medicine. We had supposed that the science of auscultation and percussion had reached perfection: indeed, that like Minerva, from the brain of Jupiter, Lænnec had presented it to the medical world fully panoplied, in all its parts, without passing through the different stages of development incident to to other sciences. But in this it seems we were mistaken.

Physical exploration of the chest, like other departments of medicine, is progressive, and has not escaped the vigilent and searching spirit of enquiry that now animates the pro fession. Our author invites attention to his study of the "intensity, pitch, and quality of sounds," as tending to increase the scope of physical exploration, and adduces examples of nice discrimination that may be made in this way. We have also some new names added to the vocabulary of the science: e. g. *vesicule-tympanitic resonance*, an exaggerated resonance sometimes found in the healthy chest; *broncho-vesicular*, or *vesiculo-tubular* respiration, *broncho-covernous* respiration. Again: *exaggerated bronchial whisper*, *whispering bronchophony*, or *bronchophonic whisper*, and *cavernous whisper*. These are new naves, which the accomplished author assures us express signs not before recognized; and we are also informed of a novel mode of auscultatory percussion, which consists in applying Camman's stethoscope near the open mouth of the patient, while percussion is made. In this way the amphoric and cracked-pot sound may often be obtained in cases in which they are not otherwise appreciable.

Finally, Prof. Flint favors us with an outline of his system of teaching auscultation and percussion to private classes at the bed side, and, in our opinion, there are few in any country more competent to give such instruction. And, from personal experience, we are satisfied that this clinical (bed-side) mode is the *only* successful one of imparting knowledge upon this important and interesting subject. Teaching it *ex-cathedra*, is simply time thrown away, and disgusts the student with a subject he cannot understand.

To a man reasonaably familiar with physical exploration, practically, the work before us is a mine of wealth. A certain amount of this kind of knowledge is necessary to its proper appreciation. Like everything from the pen of the gifted author, the subject of this work is treated to exhaustion: nothing is left to be added, and no other source of information need be consulted upon any point treated.

D. C. O'K.

